# Benefits of a one health approach: An example using Rift Valley fever

**DOI:** 10.1016/j.onehlt.2018.01.001

**Published:** 2018-01-11

**Authors:** Melinda K. Rostal, Noam Ross, Catherine Machalaba, Claudia Cordel, Janusz T. Paweska, William B. Karesh

**Affiliations:** a EcoHealth Alliance, 460 W 34th St FL 17, New York, NY 10001, USA; b ExecuVet (PTY) Ltd, 8 Roderick Crescent, Noordhoek, Bloemfontein, Free State, South Africa; c Centre for Emerging Zoonotic and Parasitic Diseases, National Institute for Communicable Diseases, Modderfontein Road 1, Gauteng, South Africa

**Keywords:** One health, Study design, Resource efficiency, Public health, Epidemiology, Outbreak investigation

## Abstract

One Health has been promoted by international institutions as a framework to improve public health outcomes. Despite strong overall interest in One Health, country-, local- and project-level implementation remains limited, likely due to the lack of pragmatic and tested operational methods for implementation and metrics for evaluation. Here we use Rift Valley fever virus as an example to demonstrate the value of using a One Health approach for both scientific and resources advantages. We demonstrate that coordinated, *a priori* investigations between One Health sectors can yield higher statistical power to elucidate important public health relationships as compared to siloed investigations and post-hoc analyses. Likewise, we demonstrate that across a project or multi-ministry health study a One Health approach can result in improved resource efficiency, with resultant cost-savings (35% in the presented case). The results of these analyses demonstrate that One Health approaches can be directly and tangibly applied to health investigations.

## Highlights

• Tangible benefits were seen with a One Health approach to health investigations.
• An *a priori* One Health investigation design can increase the statistical power.
• A 35% cost-savings was achieved through One Health resource efficiency.

## 1 Introduction

One Health, the integrated approach to management and understanding of human, animal and environmental determinants of disease, has been promoted by international institutions as a platform to improve public health outcomes [1]. The Global Health Security Agenda specifically identifies One Health as integral to achieving health security against infectious disease threats. Existing economic analyses of One Health approaches are frequently conducted at a global scale [2]. Analysis by the World Bank suggests that given the high economic and health burden of zoonotic diseases, strengthening human and veterinary health capacity to facilitate One Health approaches at the country level for disease prevention and control can yield a high return on investment (upwards of $30 billion per year) [2].

Despite strong overall interest in One Health, country-, local- and project-level implementation remains limited, likely due to the lack of pragmatic and tested operational methods for implementation and metrics for evaluation [3], [4]. Here, we contribute to closing this gap by examining an example of a One Health approach to understanding the epidemiology of Rift Valley fever (RVF) virus in Sub-Saharan Africa. Using simulations based on recent published reports of RVF virus (RVFV) exposure, we illustrate how a One Health platform improves the probability of detecting associations that could be important for improving public health. Using cost data from our ongoing epidemiological study on RVFV, we also demonstrate the increased resource efficiency of joint human-animal studies.

Rift Valley fever virus is a vector-borne zoonosis that occurs as periodic yet severe outbreaks, impacting the health of people, their livestock, and socio-economic outcomes. Outbreaks are associated with periods of greater than average rainfall, but cannot easily be predicted, despite the remote warning systems that have been developed [5]. This interaction between the environment, animal health and human health indicates that it is an ideal candidate for the application of a One Health approach to improve the understanding of RVF epidemiological dynamics and inform risk mitigation or control measures.

## 2 Statistical power of one health approaches

In understanding the epidemiology of infectious diseases, the application of One Health methods is often executed as a *post-hoc* comparison of disease outcomes in people and animals. However, associations may be missed by the analysis of results collected without the use of an *a priori* One Health design. To illustrate this, we simulated a human-cattle RVFV infected system based on data designed to emulate data from recent studies [6], [7]. We generated two distributions of cattle and human seroprevalence. The first represents the spatial distribution of RVFV seropositivity across villages in a region, with time held constant. The second represents the change in RVFV seroprevalence in one area over a multi-year period. Over each of these outputs, we then simulated two approaches to establishing associations between human and livestock seroprevalence: a One Health surveillance system with joint human-animal sampling at the same time and place, and a *post hoc* analysis of human and animal surveillance conducted at different points in time and space (Box 1).

Box 1 **Models of joint human-livestock RVFV circulation.**

To simulate *a priori* and *post hoc* studies of associations of RVFV seroprevalence in humans and livestock, we first constructed “true” simulated data sets based on published data.

For our time series model, we resampled multi-year time-series data of cattle RVFV seroprevalence from Metras et al. [7] to monthly steps and constructed a time series of human seroprevalence assuming human seroprevalence increased weakly in months following circulation in cattle, with added noise. From this series, we simulated studies using linear regression to compare the rate of seroprevalence change sampled every four months in humans and cattle, using the same points for the One Health study and offsetting human sampling from cattle by 2 months in the *post hoc* study.

For the spatial model, we used data from Olive et al. [6] on the spatial distribution of RVFV seroprevalence in Madagascar and simulated human seroprevalence assuming a noisy linear relationship at each sampling point. For the One Health study, human and cattle seroprevalence was compared *via* logistic regression of 30 points with both human and cattle data. For the *post hoc* study, 30 points each of human and cattle data were selected independently and only overlapping points (n = 9) were used.

Code and data use for simulations and analyses may be found at: https://doi.org/10.5281/zenodo.821222.

Alt-text: Box 1

**Fig. 1:**
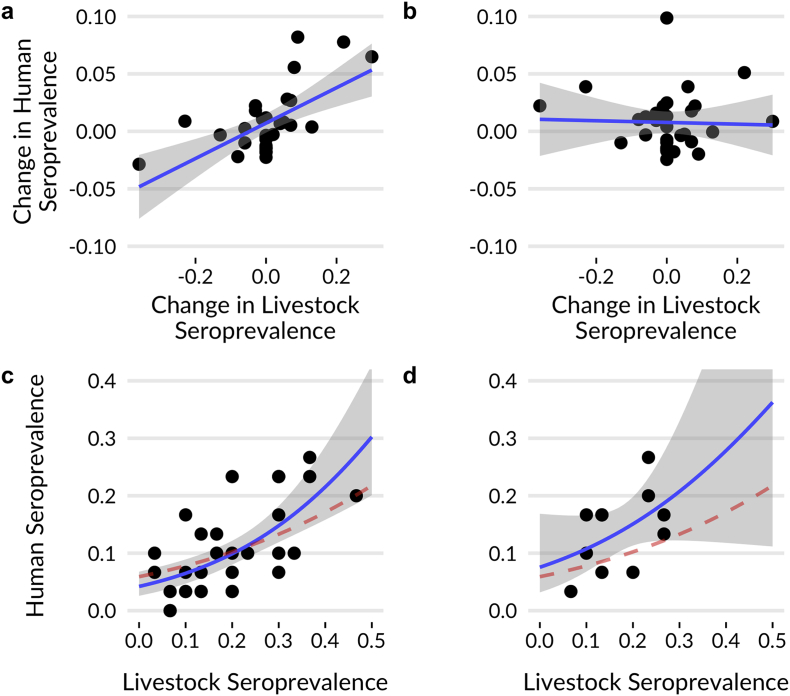
Results from simulated surveillance studies using *a priori* One Health (left) and *post-hoc* siloed (right) approaches. Top row: Results from temporal simulations. Sampling humans and livestock concurrently has the statistical power to identify a relationship between seroprevalence over time (a), the relationship is missed when sampling occurs at different times (b). Bottom row: Results from spatial simulations. Sampling humans and livestock at the same location (c) yields sufficient power to estimate the true relationship in seroprevalence (red dotted line), while relying on fewer points where co-sampling is coincident in a post-hoc study does not (d). (For interpretation of the references to colour in this figure legend, the reader is referred to the web version of this article.)

Our simulations demonstrate that the One Health sampling approach can detect associations that are missed in non-simultaneous study designs (Fig. 1). In the spatial simulation, sampling jointly in the same locations allows paired comparisons between populations at each site, revealing spatial associations of human and cattle RVFV seroprevalence (Fig. 1c). Sampling humans and cattle at different locations results in fewer effective sampling points and no ability to detect the association (Fig. 1d).

These simulations are based on real data from published studies of RVFV seroprevalence, and represent analyses frequently used to assess risk in public health (*e.g.* logistic regression). From a study design perspective, the results of the simulations are not surprising; their value instead lies in the use of these models to demonstrate the power of a One Health design to maximize statistical power, and the weakness of association from siloed investigations analysed *post hoc*. While there are certainly many examples of correlations found during *post-hoc* One Health investigations, important relationships certainly lie undiscovered with these approaches. An *a priori* One Health investigation will improve epidemiological risk assessment.

## 3 Resource efficiency from one health

One Health approaches may also improve resource efficiency. The Understanding Rift Valley Fever in Republic of South Africa Project is a U.S. Defense Threat Reduction Agency-funded study that is simultaneously evaluating the following factors: weather/climate, vegetation, soil, vector populations, ruminant host exposures and human risk factors. Transport costs for the One Health epidemiological study of RVFV in South Africa were evaluated between October 2014–January 2016. During this time, two field vehicles were used for the vegetation, soil, animal health and human health investigations. A vehicle log was used to determine the number of trips, the frequency of use by each investigation and identify vehicle sharing. Costs were estimated for each investigation based on receipts for transportation-related purchases (*e.g.* diesel, tolls, maintenance and insurance). The number of trips (total and by investigation), total cost, projected total cost (based on vehicle sharing per investigation), percent of trips shared between one or more investigation(s), percent of projected transport time and costs saved were calculated and summarized for the total time period.

The project vehicles were used in a total of 333 trips during the study period. The number of trips used to conduct work for a specific investigation varied from 71 to 149. If the vehicle had not been shared it would have made 483 trips, indicating a 31% decrease in the number of trips made for the research study due to sharing. The actual cost to the One Health project was $11,744.41 (*versus* the projected cost of making individual trips of $18,177.08), a savings of 35% or $6432.61. This savings could then be reallocated within the project to support other activities.

Our demonstrated cost savings of 35% at a project scale using actual costs from vehicle sharing within an integrated One Health program are consistent with preliminary estimates of efficiency gains reported by the World Bank, including 10–30% savings from One Health disease surveillance transportation and communication costs for low-middle income countries in low and high pathogen prevalence scenarios [2]. These estimates need to be ground-truthed by local examples of cost-sharing, such as the analysis presented here.

By cross-training staff in working with human subjects and animals the RVFV project was able to minimize team size (5 per vehicle) while maximizing the output. All of the animal handling and sampling team members were trained in ethical concerns regarding working with human subjects; informed, valid and voluntary consent; confidentiality *etc*. This improved efficiency as all members of the team had the training and awareness to assist on other work program tasks to reduce the amount of time spent on the farm. Likewise, it is important to quantify the amount of time saved for the participant when both animal and human sampling can be conducted concurrently and the project description and consent process only needs to be given to the participant once (if their animals are also being sampled), rather than needing to have separate conversations for individual human and animal sampling visits (*i.e.*, reduced opportunity cost for participation). Time savings from more rapid and accurate epidemiological associations (*e.g.*Fig. 1) may also enable efficiencies for control that can ultimately reduce outbreak burden.

Small-scale cost-effectiveness analyses can be conducted on additional budget line items. Salary analyses may show offsets from this synergistic approach that in total decreases the staffing demands per department/ministry (health, agriculture, environment) required for an investigation. A study in Chad investigating cost-savings of conducting joint vaccination campaigns for livestock and people including personnel/administrative, transportation, cold chain and other costs (not including vaccination costs) found the proportion of costs shared by the veterinary and public health sectors to be 4.1–15.1% [8]. Likewise, an integrated human and animal disease laboratory in Winnipeg, Canada estimated operational cost efficiency gains at 26% per year [2].

## 4 Conclusions

This RVFV example demonstrates both the scientific and resource advantages in coordinated, *a priori* investigations between One Health sectors as compared to siloed investigations and *post-hoc* analyses. While recognizing institutional challenges for governmental agencies to parse out the costs of their partial support of a One Health team, we call upon international funders and ministerial leaders to incorporate quantitative resource efficiency metrics as part of the evaluation of their sponsored One Health programs. Findings may demonstrate resource optimization or needs for adjustment to resource allocation for current or future support of One Health programs.

## Conflict of interest statement

The authors have no conflict of interest to declare.
